# Measuring Synchronization between Spikes and Local Field Potential Based on the Kullback–Leibler Divergence

**DOI:** 10.1155/2021/9954302

**Published:** 2021-09-09

**Authors:** Liyong Yin, Guangrong Zhang, Fuzai Yin

**Affiliations:** ^1^Department of Internal Medicine, Hebei Medical University, Shijiazhuang 050011, China; ^2^School of Information Science and Engineering, Yanshan University, Qinhuangdao 066004, China; ^3^Department of Endocrinology, The First Hospital of Qinhuangdao, Qinhuangdao 066002, China

## Abstract

Neurophysiological studies have shown that there is a close relationship between spikes and local field potential (LFP), which reflects crucial neural coding information. In this paper, we used a new method to evaluate the synchronization between spikes and LFP. All possible phases of LFP from −*π* to *π* were first binned into a freely chosen number of bins; then, the probability of spikes falling in each bin was calculated, and the deviation degree from the uniform distribution based on the Kullback–Leibler divergence was calculated to define the synchronization between spikes and LFP. The simulation results demonstrate that the method is rapid, basically unaffected by the total number of spikes, and can adequately resist the noise of spike trains. We applied this method to the experimental data of patients with intractable epilepsy, and we observed the synchronization between spikes and LFP in the formation of memory. These results show that our proposed method is a powerful tool that can quantitatively measure the synchronization between spikes and LFP.

## 1. Introduction

The information in the nervous system is distributed over a large number of neurons. In order to understand how this information is encoded, processed, and translated into action, we need to monitor the group activities of a considerable number of neurons [1, 2]. With the development of electrode manufacturing technology and microelectronics technology, multielectrode synchronous recording technology has emerged [3], which can observe the activity characteristics of multiple neuron groups simultaneously and is helpful to analyze and decode the behavioural and biological information carried in the spike train and the local field potential (LFP) [4].

The cerebral cortex encodes sensory information through the activity of neurons. This phenomenon has been widely studied in the extracellular records of conscious animals [5]. This record can capture the spike activity, which is a short-term high-frequency signal and reflects more individual activities [6]. The spikes encode the rate and temporal distribution of these binary events [5]. LFP is another component of the extracellular signal, namely, the low-frequency part of the neural signal that reflects the synaptic activity of neighbouring neurons [6–8]. Recently, LFP has attracted increasing attention in the fields of neuroscience and neural engineering. It is considered to be the sum of thousands of synaptic potential fluctuations around the recording electrode [5].

Synchronization between spikes and LFP has been observed in different cognitive functions and different brain regions, such as the prefrontal cortex [5, 9] and the hippocampus [6, 10, 11]. In recent years, many neurophysiological studies have shown that there is a close relationship between spikes and the gamma-band LFP [9, 12], and it has been shown that the phase locking between the spikes and LFP reflects vital physiological information. For instance, a close phase locking between the spikes and the gamma rhythm of LFP in the pyramidal neurons of the rat olfactory cortex was reported, and gamma oscillatory wave-like filters can control the emission time of neurons [13]. Simulation studies showed that the phase-locking relationship between spikes and the LFP rhythm may encode cognitive events [14], and the gamma rhythm of the visual cortex of rhesus monkeys can encode the contrast information of the image [15]. In the human brain, the coordinated relationship between spike emissions and the theta rhythm of LFP is closely related to the formation of memory [16]. Therefore, it is of great significance to study the relationship between spikes and LFP.

The spiking activity of many different neuron types synchronizes to the local LFP [17]. There are different measures for estimating spike-LFP synchronization, including cross correlation and coherence coefficient [18–22], phase synchronization or the phase-locking value (PLV) [23], spike field coherence (SFC) [24–27], pairwise phase consistency (PPC) [28], and spike-triggered correlation matrix synchronization (SCMS) [29, 30]. Cross correlation and coherence coefficient methods are biased towards the power and are not suitable for nonlinear and nonstationary dynamics [31]. Some of the most widely used spike-LFP synchronization measures are the PLV, the SFC, the PPC, and the SCMS. The PLV computes the magnitude of the mean phase difference between LFP and spikes. The disadvantage of this method is that it strongly depends on the spike rate [23]. The SFC is computed by comparing the magnitude of the frequency in the spike-triggered average (STA) and the average magnitude of the frequency for each LFP segment that is involved in the STA. The SFC also depends on the rates of the spikes [26]. The PPC calculates the mean spike similarity between all possible spikes over LFP phases. The shortcoming of the PPC is that the result may appear as a negative value for some cases, which cannot be justified physiologically [28]. The SCMS uses the phase of LFP in the area around the spikes to measure correlation rather than the moment of the spike's occurrence. The SCMS has the problem of a slow calculation speed [29].

In this paper, we used the modulation index (MI) algorithm to measure the synchronization between spikes and LFP, which has been employed to estimate phase-amplitude coupling (PAC) [32]. The simulation results demonstrated that the performance of this method does not depend on the total number of spikes. Additionally, it is robust to the spike noise, including extra spikes, missing spikes, and jitter noise. Moreover, the method is easy to implement with a fast running speed.

## 2. Materials and Methods

**Figure 1:**
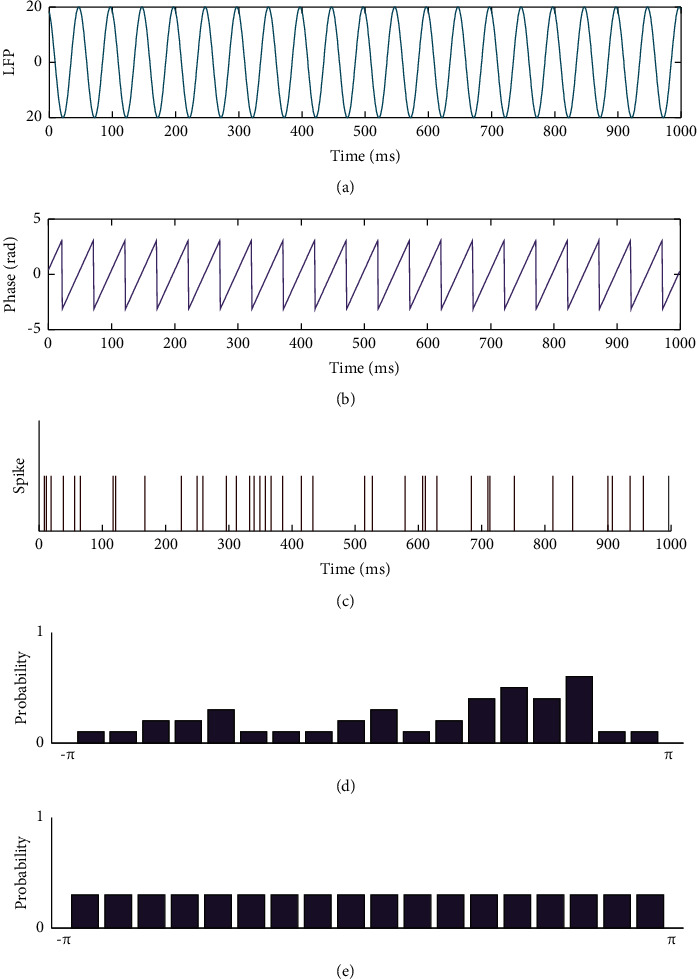
Schematic for the calculation procedure. (a) The raw data of LFP. (b) The instantaneous phase of the LFP. (c) The spike train. (d) The probability of spikes in each bin. (e) The uniform distribution of spikes in each bin.

The main idea of this new method to evaluate the synchronization between spikes and LFP is explained below. First, all possible phases of LFP from −*π* to *π* were binned into a freely chosen number of bins. Eighteen bins were adopted in this paper, corresponding to each bin of *π*/9, which was in accordance with other studies [33]. Then, we calculated the probability of the spikes falling in each bin and the information entropy corresponding to the spike distribution. Finally, we compared the spike distribution with the uniform distribution by means of the Kullback–Leibler (KL) divergence and adopted the MI [32] to measure the synchronization relationship between spikes and LFP. Part of the schematic diagram of the calculation process is shown in Figure 1. More details of the algorithm are provided in the following section.

### 2.1. LFP Phase Extraction

First, zero-phase-shift filters were used to filter the LFP frequency band, and then Hilbert transform was used to extract the instantaneous phase of the LFP signal. For the signal *x*(*t*), the analytical signal *z*(*t*) is a complex function of time *t*, which is defined as

(1) $$\begin{array}{c} z\left(t\right)=x\left(t\right)+i\tilde{x}\left(t\right)=a\left(t\right)e^{\varphi \left(t\right)}, \end{array}$$

where *a*(*t*) is the instantaneous envelope of *z*(*t*), *φ*(*t*) is the instantaneous phase of *z*(*t*), and $\tilde{x}\left(t\right)$ is the Hilbert transform of *x*(*t*). Specifically, $\tilde{x}\left(t\right)$ is defined as

(2) $$\begin{array}{c} \tilde{x}\left(t\right)=\frac{1}{\pi }P.V.\overset{+\infty }{\underset{-\infty }{\int }}\frac{x\left(\tau \right)}{t-\tau }d\tau , \end{array}$$

where *𝒫*.*𝒱*. indicates that the integral is taken in the sense of the Cauchy principal value [34]. Thus, the instantaneous phase of the LFP signal can be obtained by

(3) $$\begin{array}{c} \phi \left(t\right)=\arctan\left(\frac{\tilde{x}\left(t\right)}{x\left(t\right)}\right). \end{array}$$

### 2.2. MI Calculation

The LFP signal was defined as *x*(*t*). The instantaneous phase *φ*(*t*) was extracted from *x*(*t*), and all possible phases of LFP from −*π* to *π* were first binned into a freely chosen number of bins. The probability of spikes falling in each bin of LFP was calculated, denoted as *P*(*j*):

(4) $$\begin{array}{c} P\left(j\right)=\frac{n_{j}}{\sum_{j=1}^{N}n_{j}}, \end{array}$$

where *P*(*j*) represents the probability of the spikes appearing in the corresponding bin of the LFP phase, *n*_*j*_ is the number in one bin, *j* is the running index for bins, and *N* is the total number of bins. With these calculations, the data of the LFP-spike distribution were obtained.

Then, the Shannon entropy was calculated. The Shannon entropy is a measure of the inherent information of a variable. If the Shannon entropy is not maximal, there is redundancy and predictability in the variable. If spikes appear randomly in the LFP and are not synchronized, the Shannon entropy is maximal. The Shannon entropy is calculated by the following formula:

(5) $$\begin{array}{c} H\left(P\right)=-\sum_{j=1}^{N}P\left(j\right)\log\;P\left(j\right). \end{array}$$

Spike-LFP synchronization is defined as a distribution that clearly deviates from a uniform distribution. The KL divergence is an effective tool to measure the difference between two distributions which has been used in many studies [32, 35, 36]. It is related to the Shannon entropy and is calculated by

(6) $$\begin{array}{c} \text{KL}\left(U,P\right)=\log\;N-H\left(P\right), \end{array}$$

where *U* is the uniform distribution, *P* is the data distribution, and *N* is the total number of bins. *H*(*P*) is the Shannon entropy mentioned in equation (6), and log  *N* is the maximum possible entropy value, which represents a uniform distribution (when *P*(*j*)=1/*N*, applicable to all bins). Therefore, we define the MI by dividing the KL divergence of the observed spike distribution (*P*) and the uniform distribution (*U*) by log  *N*, and the final MI is given by

(7) $$\begin{array}{c} \text{MI}=\frac{\text{KL}\left(U,P\right)}{\log\;N}, \end{array}$$

where KL(*U*, *P*) is the KL divergence according to equation (6) and *N* is the total number of bins [31, 32].

If spikes are uniformly distributed over the phase of LFP (i.e., *P*=*U*, which means there is no synchronization between the spikes and LFP), then MI=0. As *P* moves farther and farther away from *U*, the KL divergence increases gradually; when MI=1, all spikes exist in a certain bin phase of the LFP, and there are no spikes in the other bins. Then, there is a strong synchronous relationship between the spikes and LFP.

### 2.3. Simulated Data

**Figure 2:**
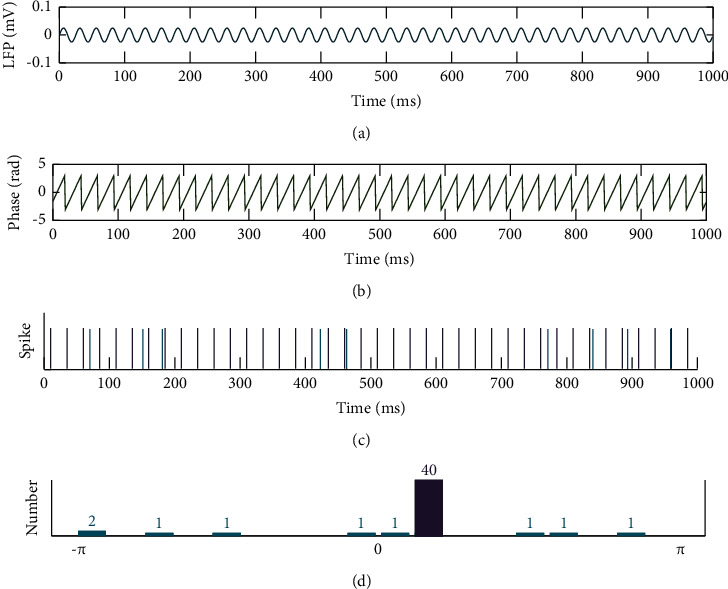
An example of synchronous and asynchronous spikes. (a) The simulated LFP. (b) The phase of LFP. (c) Synchronous spikes (purple lines) and asynchronous spikes (cyan lines). (d) The number of spikes in each phase bin.

The LFP signal used in the simulations was represented by the superposition of several sine waves with different frequencies, different amplitudes, and different phases. The frequency ranged from 30 to 80 Hz with a step of 1 Hz, thereby focusing on the gamma band in the LFP. The amplitude of the sine wave was inversely proportional to its corresponding frequency [16], and the phase of the signal was randomly selected in the range of [−*π*, *π*]. Spikes were generated by inserting 0 and 1 in the time interval, where 0 denoted no spikes and 1 indicated the firing time of spikes. Each spike was either synchronous or nonsynchronous with the phase of LFP. Synchronized spikes fired by simulated individual neurons were located at some certain times (there are some cycles of the summed LFP waveform between two spikes), while nonsynchronous spikes appeared randomly. The numbers of synchronous spikes were denoted by *s*_*y*_; the numbers of nonsynchronous spikes were expressed by *s*_*u*_. The total number of spikes *s*_*n*_ was the sum of synchronous and asynchronous spikes, i.e., *s*_*n*_=*s*_*u*_+*s*_*y*_. The parameter controlling the synchronization strength was defined as the ratio of the number of synchronization spikes to the total number of spikes, which was calculated by *R*=*s*_*y*_/*s*_*n*_. *R*=1 means perfect synchronization, and *R*=0 means complete nonsynchronization [29]. An example of the distribution of synchronous and asynchronous spikes is shown in Figure 2.

### 2.4. Real Data

In this paper, the MI method was applied to the data collected by the Centre for Systems Neuroscience (University of Leicester, Leicester LE1 7RH, UK). The data were recorded from 21 experimental sessions of six patients with pharmacologically intractable epilepsy (all right-handed, four males, aged 23–56 years). Patients were implanted with chronic depth electrodes for 7–10 days at King's College Hospital in London (UK) to identify the epileptic foci that may be surgically resected [37]. All patients agreed in writing to participate in this study and were approved by King's College Hospital Research Ethics Committee. There was a total of nine microwires at the end of each electrode probe, including eight active recording channels and one (low impedance) reference. The electrodes were implanted bilaterally in the hippocampus (24 probes) and amygdala (12 probes). The locations of the inserted electrodes fully met the clinical criteria and were verified by magnetic resonance imaging (MRI) or computed tomography (CT) combined with preoperative MRI recordings.

A simple visual task was used to identify responsive stimuli. When the subject sat in front of a laptop, a group of about 100 stimuli was presented on the computer and was displayed six times, each in pseudorandom order, by using a block design. At the beginning of each trial, there was a fixation cross on the screen for 500 ms, followed by a picture displayed for 1000 ms. After the screen turned black, the patient had to press a button to answer whether or not there was a person in the picture just shown. The intertrial interval varied randomly between 600 and 800 ms. These “screening sessions” typically lasted about half an hour. The images included familiar people, animals, landmarks, and friends and family of the patient.

Once it had been determined which picture(s) triggered the firing of which neuron, we performed follow-up sessions in which we used a subset of about 15 stimuli from the screening session (including all those that elicited a response), but each of these images was displayed 25–35 times in pseudorandom order.

**Figure 3:**
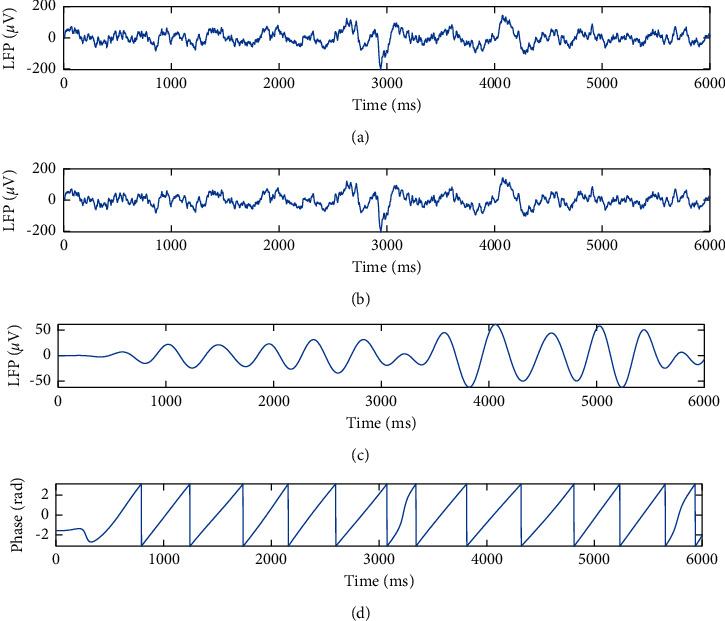
Extracting process for the LFP recorded in real experiments. (a) The raw data. (b) Downsampled data. (c) Theta-band LFP. (d) The phase of theta-band LFP.

The collected data were processed offline, and recorded neuron spikes were identified by extracting signals higher than 300 Hz. Subsequently, Wave_clus was used for spike detection and sorting. On the other hand, the raw data were downsampled to 1.5 kHz and then they were further filtered between 3 and 6 Hz (zero-phase elliptic filter) to obtain the theta-band LFP. We discarded the channels showing high-frequency noise by calculating the power spectrum of the recorded channels. The single-trial LFP traces were extracted from 1 s before to 2 s after stimulus onset [37–39]. The extracting process of theta-band LFP is shown in Figure 3.

## 3. Results

### 3.1. Application to Artificial Data

#### 3.1.1. The Influence of Synchronization Strength on the Algorithm

**Figure 4:**
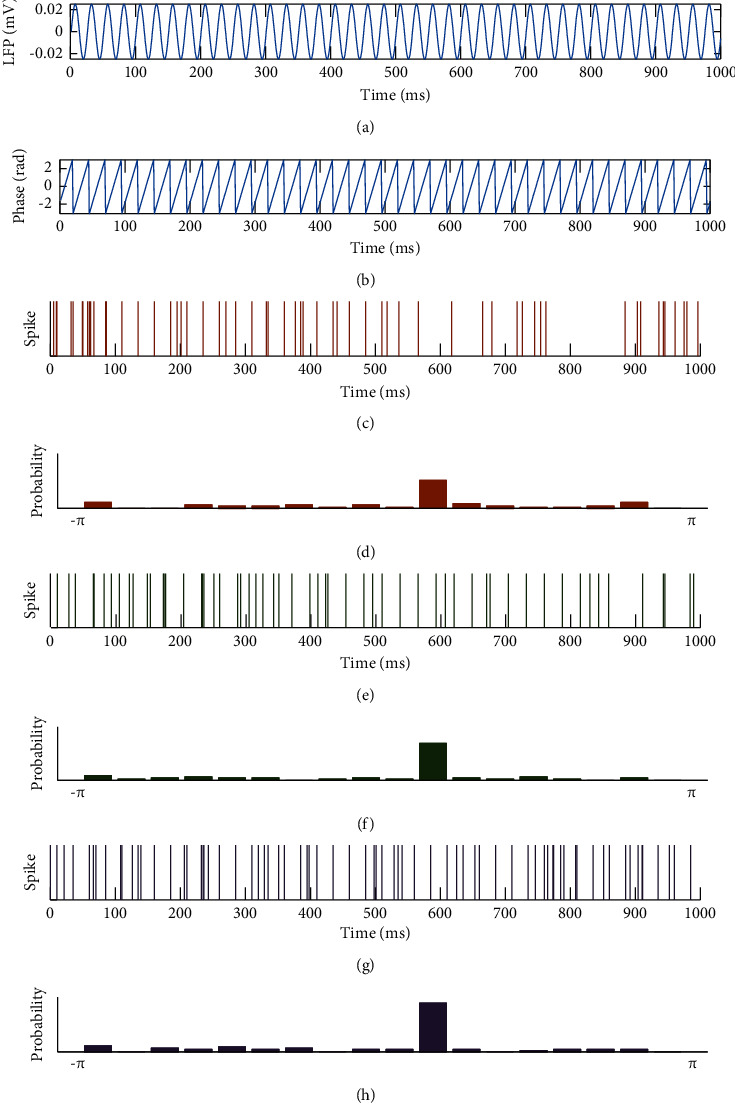
Examples of LFP and spikes with different synchronization strengths. (a) The simulated LFP. (b) The phase of LFP. (c, e, g) The spike firing times (*R* = 0.3, 0.5, and 0.7). (d, f, h) The probability of spikes in each bin.

**Figure 5:**
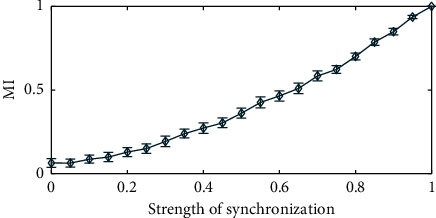
Variation in the MI values with synchronization strength.

We demonstrated the variation in estimated MI values with the strength of the spike-LFP synchronization, which ranged from 0 to 1 with a step interval of 0.05. The duration of LFP used in the simulation was 100 s, and the sampling rate was 1 kHz. These parameter settings were also used in the subsequent simulation sections. The distribution difference of spike-LFP with different synchronization strength is shown in Figure 4. We carried out 100 trials on the studied parameters (synchronization strength), and the simulation results are shown in Figure 5. It can be seen that the MI was a normalized quantity. With the increase of spike-LFP synchronization strength, the MI value gradually increased to 1.

#### 3.1.2. MI Dependence on Spike Number

**Figure 6:**
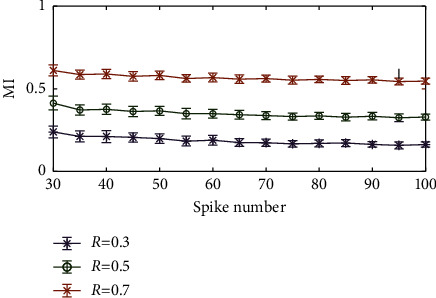
Effect of the total spike number on the MI value.

In this section, we discussed the dependence of the MI method on the number of spikes. For the initial LFP, the initial number of spikes we used was 30. To observe the influence of the spike number on the performance of the algorithm, we gradually increased the spike number from 30 to 100 with a step of 5. The strength of the simulated spike-LFP synchronization was set to 0.3, 0.5, and 0.7. We ran 100 trials for each set of parameters under study (the length of data). Results of this analysis are shown in Figure 6. We found that the MI value hardly changed with the increase in spike number, which was crucial when we compared different experimental conditions.

#### 3.1.3. Influence of Spike Noise

The influence of spike noise on the algorithm could not be ignored when we calculated the spike-LFP synchronization relationship. Three types of noise were considered: jitter noise (a shift in spiking time), missing spikes (false negatives), and extra spikes (false positives) [29]. The noise generated by spikes was mainly associated with spike detection and classification. For example, extra and missing spikes may be caused by unreasonable threshold settings during spike detection. Of course, these two types of noises also occurred in the process of spike classification. Another type of noise occurred in the process of spike acquisition or spike alignment, i.e., jitter noise. In this section, we discuss the impact of the three types of noise generated by spikes. For each type of noise, the synchronization strength was *R* = 0.3, *R* = 0.5, and *R* = 0.7.

**Figure 7:**
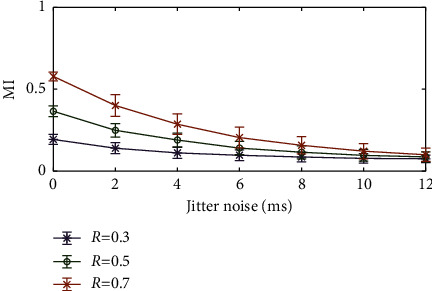
Effect of jitter noise in the spike trains on the MI.

First, we considered the effect of jitter noise [40] on the results. The number of spikes in the simulation was set to 50. The specific operation of adding jitter noise assumed that the spikes were emitted at time *T*, the length of the time window of the jitter noise was *t*, and the strength of the jitter noise was quantified by the interval length. The jitter noise caused the spikes emitted at time *T* to appear randomly in the time range of [*T* − *t*, *T* + *t*]. The parameters (jitter noise) of each group were averaged across 100 trials, and the simulation results are shown in Figure 7. These simulation results showed that the MI value decreased as the jitter interval increased, but within 10 ms, we could still distinguish the different strengths of the spike-LFP synchronization. These results indicate that the MI algorithm can provide meaningful results when comparing the synchronization strength between different neurons and LFP.

**Figure 8:**
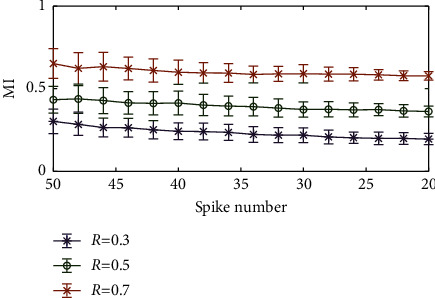
Effect of missing spikes in the spike trains on the MI.

Second, we studied the influence of missing spikes on the results of the algorithm. Similarly, the initial number of spikes in the simulation was set to 50. The specific operation was that some spikes were randomly removed from the original spike trains. The number of missing spikes ranged from 2 to 30, with a step interval of 2. One hundred trials were carried out on each group of parameters (missing spikes). The simulation results based on the average of 100 trials are shown in Figure 8. It can be seen that the MI value was less affected by the missing spike noise because randomly removing some spikes did not essentially change the synchronization strength between the spike train and LFP. The MI was not affected by the number of spikes, so it can adequately resist the missing spike noise.

**Figure 9:**
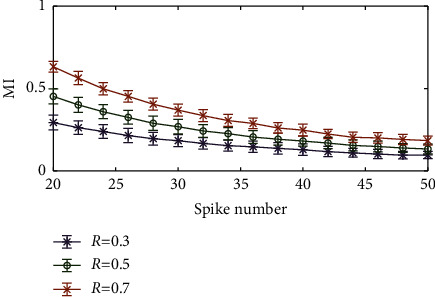
Effect of extra spikes in the spike trains on the MI.

Finally, the impact of extra spikes [41] on the results was considered. The specific operation entailed the random insertion of extra spikes into the original spike trains. In the simulation, the original number of spikes was 20. The number of extra spikes ranged from 2 to 30, with a step interval of 2. We ran 100 trials for each set of parameters under study (extra spikes). The results of this analysis are shown in Figure 9. It can be seen that MI decreased with the increase in the number of spikes. This was because the spikes increased randomly, which is equivalent to the decrease in the synchronization strength. As a result, as MI decreased, we could still easily distinguish different synchronization strengths.

### 3.2. Application to Real Data

In this study, we investigated the relationship between the neural activities in medial temporal lobe (MTL) and memory. For each response, we considered the spikes in two time windows, “baseline” (time window in the baseline period) and “response” (time window starting at the spike response latency). At each spike time, we used the angle of the Hilbert transform of single-trial LFP filtered in the theta band (3–6 Hz) to calculate the instantaneous phase [39].

**Figure 10:**
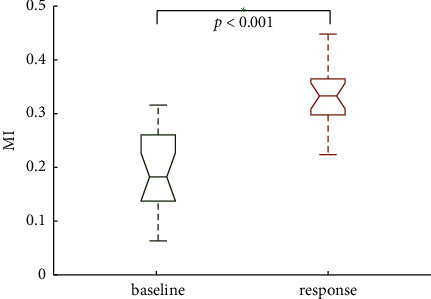
The results applied to real data.

A recent study in humans also strengthened the link between MTL and memory function by showing that the phase locking between spiking and theta activity in this area during encoding predicted memory success [16]. In this paper, we used our proposed method to investigate the synchronization relationship between the theta rhythm of LFP and spikes in the human MTL in the memory process. We selected 10 of 19 groups of experiments to calculate MI, and the specific results are shown in Figure 10. We found that the MI value in the “response” was significantly higher than that at the “baseline.” This implies that the spikes and LFP of recorded neurons show obvious synchronization during memory (*p* < 0.001, Kruskal–Wallis test).

## 4. Conclusions

This paper studied the synchronization between spikes and LFP by using the MI algorithm. We showed that the MI algorithm was basically not affected by the total number of spikes. The method was simple to calculate and can resist spike noise arising from jitter, extra spikes, and missing spikes. Therefore, the MI algorithm used in this paper is a robust algorithm that can quantitatively analyze the synchronization between spikes and LFP. By applying the method to neuronal data recorded from patients with epilepsy, we showed that the spike-LFP synchronization in the “response” was higher than that in the “baseline” by using the Kruskal–Wallis test, and we demonstrated that spike-LFP synchronization can be used to explore the connection between the MTL and memory function.

## Data Availability

The data used to support the findings of this study are available at https://www2.le.ac.uk/centres/csn/data.
